# Age-Related DNA Methylation in Normal Kidney Tissue Identifies Epigenetic Cancer Risk Susceptibility Loci in the ANKRD34B and ZIC1 Genes

**DOI:** 10.3390/ijms23105327

**Published:** 2022-05-10

**Authors:** Jürgen Serth, Inga Peters, Bastian Hill, Tatjana Hübscher, Jörg Hennenlotter, Michael Klintschar, Markus Antonius Kuczyk

**Affiliations:** 1 Department of Urology and Urologic Oncology, Hannover Medical School, 30625 Hannover, Germany; peters.inga@mh-hannover.de (I.P.); bastian.hill@googlemail.com (B.H.); tatjanahuebscher@gmail.com (T.H.); kuczyk.markus@mh-hannover.de (M.A.K.); 2 Department of Urology, Eberhard Karls University of Tuebingen, 72076 Tuebingen, Germany; joerg.hennenlotter@med.uni-tuebingen.de; 3 Department of Legal Medicine, Hannover Medical School, 30625 Hannover, Germany; klintschar.michael@mh-hannover.de

**Keywords:** age-related methylation, age-dependent methylation, renal cell cancer, epigenetic, cancer susceptibility locus, cancer development, DNA-methylation, age signature, cancer diagnosis, cancer prediction

## Abstract

Both age-dependent and age-independent alteration of DNA methylation in human tissues are functionally associated with the development of many malignant and non-malignant human diseases. TCGA-KIRC data were biometrically analyzed to identify new loci with age-dependent DNA methylation that may contribute to tumor risk in normal kidney tissue. *ANKRD34B* and *ZIC1* were evaluated as candidate genes by pyrosequencing of 539 tissues, including 239 normal autopsy, 157 histopathologically tumor-adjacent normal, and 143 paired tumor kidney samples. All candidate CpG loci demonstrated a strong correlation between relative methylation levels and age (R = 0.70–0.88, *p* < 2 × 10^−16^) and seven out of 10 loci were capable of predicting chronological age in normal kidney tissues, explaining 84% of the variance (R = 0.92). Moreover, significantly increased age-independent methylation was found for 9 out of 10 CpG loci in tumor-adjacent tissues, compared to normal autopsy tissues (*p* = 0.001–0.028). Comparing tumor and paired tumor-adjacent tissues revealed two patient clusters showing hypermethylation, one cluster without significant changes in methylation, and a smaller cluster demonstrating hypomethylation in the tumors (*p* < 1 × 10^−10^). Taken together, our results show the presence of additional methylation risk factors besides age for renal cancer in normal kidney tissue. Concurrent tumor-specific hypermethylation suggests a subset of these loci are candidates for epigenetic renal cancer susceptibility.

## 1. Introduction

Specific alterations in DNA methylation (DNAme) in human tissues and concurrent changes in the epigenetic regulation of gene expression are associated with a substantial number of malignant and non-malignant human diseases. Thus, elucidation of disease-specific DNAme patterns could improve the early detection, prognosis, and functional characterization of related pathogenic processes [1]. The epigenetic stem cell model of cancer connects epigenetic alterations, including DNAme, and stem cell gene silencing by Polycomb repressor complex 2 (PRC2) [2]. Accumulated hypermethylation and hypomethylation of Polycomb repressor target genes have been reported as hallmarks of malignant cellular transformation, explaining why age and the related number of cell divisions are the most important cancer risk factors (ibid). Moreover, interaction with genetic factors and external factors, including environmental and lifestyle effects, have been described to modify DNAme in a tissue- and gene-specific manner, providing a link between known cancer risk factors and molecular changes in cancer development [3].

Renal cell cancer (RCC) accounts for 3% and 5% of cancer diagnoses in men and woman, respectively, and approximately 25% of patients exhibit distant metastasis at the time the disease is detected [4]. Epidemiological analyses have identified age, obesity, and smoking as the most important risk factors for the development of the disease [5,6].

Epigenetic alterations have been reported to account for more than 60% of RCC risk [7], and a substantial number of reports have described an association of DNAme loci with the clinical stage, state of metastasis, prognosis, and therapeutic response of RCC [8].

Studies of DNAme at specific loci and the association with age or other RCC risk factors are relatively sparse, likely because analyses require normal human kidney samples from tissue donors with documented exposure to RCC risk factors. However, qualitative assessment of age-dependent methylation of selected genes in normal tissues has been described [9]. When comparing normal samples and tumor-adjacent normal (adN) samples, we found age-dependent DNAme of the *SFRP1* loci, which was further increased in adN tissue samples, corresponding to odds of approximately 13 in interquartile analysis [10]. Recently, we reported age-dependent DNAme of the *TBR1* loci [11] biometrically identified as a candidate region using a subset of adN data from the KIRC study branch of The Cancer Genome Atlas (TCGA) network study [12]. Interestingly, a comparison of cases and controls indicated an association of increased DNAme with tissue adiposity, providing a possible molecular link to the known epidemiological risk factor [11].

Here, we evaluated the Ankyrin Repeat Domain 34B (*ANKRD34B*) and Zic Family Member 1 (*ZIC1*) loci that were also biometrically identified as candidates in the KIRC-based adN analysis. The ANKRD34B protein is a cytosolic phosphoprotein described as being involved in the differentiation of mouse bone marrow cells [13]. The Human Protein Atlas (proteinatlas.org) reports high mRNA expression in prostate cancer and RCC, and immunopositivity has been detected in the cytoplasm of normal renal tubular cells, although RCCs tend to have a lack of signals [14]. Published data on the role of *ANKRD34B* in human disease is sparse. A biometrical study aiming to identify a biomarker signature for patients with Alzheimer’s disease reported a differentially methylated site in *ANKRD34B* [15], but increased mRNA expression has been reported in prostate carcinoma [16].

The ZIC1 protein belongs to the C2H2-type zinc finger family of proteins and exhibits transcriptional activator function with involvement in organogenesis of the central nervous system [17]. DNAme analyses of *ZIC1* have been carried out to identify epigenetic silencing in colorectal cancer [18], gastric cancer [19], hepatocellular cancer [20,21], thyroid cancer [22], mesenchymal proliferation [23], malignant pleural mesothelioma [24], and myoblast development [25]; to detect precancerous alterations of the cervix [26,27,28]; to allow noninvasive detection of endometrial cancer [29] and gastric cancer [30,31]; to achieve clinical stratification of cervical cancer [32], anal cancer [33], and ovarian cancer [34]; and to assess the aggressiveness and progression of head and neck squamous cell carcinomas [35], high-grade non-invasive bladder cancer [36], gastric cancer [37], and ovarian cancer [38].

The *ZIC1* loci was among the 2623 differentially methylated CpG sites annotated to 1405 genes in a meta study of epigenome-wide association studies (EWASs) comparing blood-derived DNA from smokers and non-smokers [39]. Interestingly, the *ZIC1* locus was one of only three loci showing increased methylation in smokers in both blood and postmortem samples of the nucleus accumbens [40], indicating that *ZIC1* methylation could be affected by lifestyle factors in various cell types. Both *ANKRD34B* and *ZIC1* have been reported in a pan-cancer analysis to be targets of PRC2, with hypermethylated CGIs [41].

Here, we showed that DNAme of candidate loci *ANKRD34B* and *ZIC1* strongly correlates with age and allows the estimation of chronological age with an acceptable error rate. Interestingly, a subgroup of tissues had significantly elevated levels of DNAme in adN samples that were not explained by age. In view of concurrent tumor-specific hypermethylation, our results suggested the corresponding loci as candidates for epigenetic renal cancer susceptibility.

## 2. Results

### 2.1. Biometrical Analysis

**Figure 1:**
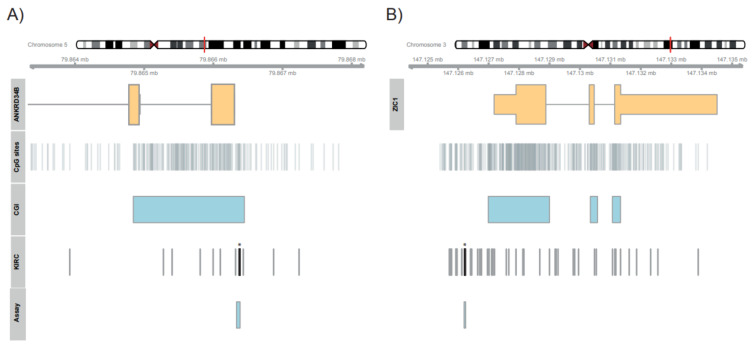
Genomic organization of candidate regions of ANKRD34B (**A**) and ZIC1 (**B**) showing exons (thick orange rectangular boxes), genomic regions corresponding to UTRs (thinner orange boxes, only for ZIC1), CpG islands (CGI), all (CpG sites) and KIRC annotated CpG sites (KIRC), location of candidate sites (Candid.) and pyro sequencing assay (Assay).

Pearson correlation analysis of TCGA-KIRC methylation data and tissue donor age revealed coefficients of correlation of 0.66, 0.64, and 0.63 for the cg25316339, cg16181396, and cg218002332 loci, respectively (all *p* < 2 × 10^−16^). Inspection and measurement of control DNA and a pilot cohort and indicated that both candidate regions are technically amenable for evaluation by pyrosequencing (Figure 1; see Supplementary Figure S1 for primary data). The genomic positions of the assays and measurable CpG positions are presented in Table 1.

### 2.2. ANKRD34B and ZIC1 Loci Show a Strong Correlation with Age in Normal Kidney Tissues

**Figure 2:**
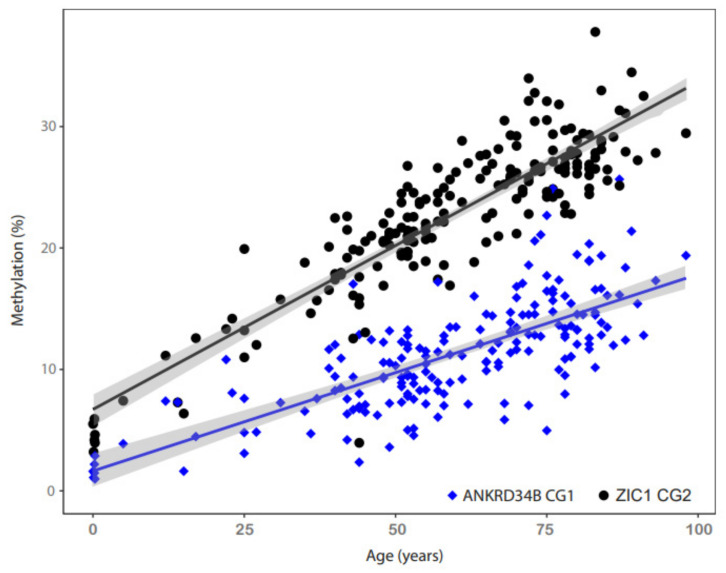
Age dependent methylation of the *ANKRD34B* (blue rhombus shaped symbols and blue regression line) and *ZIC1* (black solid circles and black regression line) CpG sites CG1 and CG2 exhibiting maximum R-values in Pearsons correlation analysis as specified in Table 1. Grey shaded areas indicate the 95% confidence intervals for regression lines.

Evaluation of candidate CpG loci methylation by pyrosequencing analysis of the normal tissue cohort demonstrated that 10 (100%) of the loci exhibited strong age-dependent methylation. Coefficients of correlation of 0.61–0.88 (all *p* < 2 × 10^−16^) were obtained in the Pearson correlation analysis, confirming all of the biometrical candidate loci (Table 1). Linear regression analysis revealed maximum increases in the methylation per year for the CG1 site of *ANKRD34B* (slope = 0.16; 95% CI 0.15–0.19) and the CG2 site of *ZIC1* (slope = 0.26, 95% CI 0.24–0.28), corresponding to an expected 100-year lifetime-accumulated relative methylation change of 16% and 26% in normal tissues, respectively (Figure 2). Linear regression lines and confidence intervals (Figure 2, grey shaded areas) also indicate a significantly increased rate of methylation for the *ZIC1* CG2 site compared to the *ANKRD34B* CG1 site in normal tissues.

Analysis of age-dependent methylation in adN tissues revealed significant relationships for 3 out of 10 CpG sites analyzed, resulting in R = 0.29 (*ANKRD34B* CG3, *p* = 0.02), 0.36, and 0.51 (*ZIC1* CG2 and -CG3, respectively, *p* < 0.001). No significant association of CpG candidate site methylation and age was found in tumor tissues (R = 0.08–0.18).

### 2.3. Chronological Age Prediction of Normal Kidney Tissues

We recently reported the identification of age-related methylation of *TBR1* CpG loci in kidney tissues [11]. One of these loci was described previously to be usable for methylation-based donor age determination via the measurement of saliva samples [42]. Whether *ANKRD34B* and *ZIC1* loci contribute to age determination of kidney tissues was analyzed by random training, test cohorts, and linear regression, including a stepwise selection of variables and a 20-fold cross-validation. Methylation of 7 out of 10 (70%) loci, including CG1-CG3 from the *ZIC1* region and CG1-CG4 from the *ANKRD34B* region, was identified as a significant model parameter to predict age in the unknown test cohort. A mean average error (MAE) of 6.9 years and an R value of 0.92, explaining 84% of the observed variance, were obtained for age prediction (Supplementary Figure S2).

### 2.4. Age-Independent Increase of ANKRD34B and ZIC1 Methylation in Normal High-Risk Tissues

**Figure 3:**
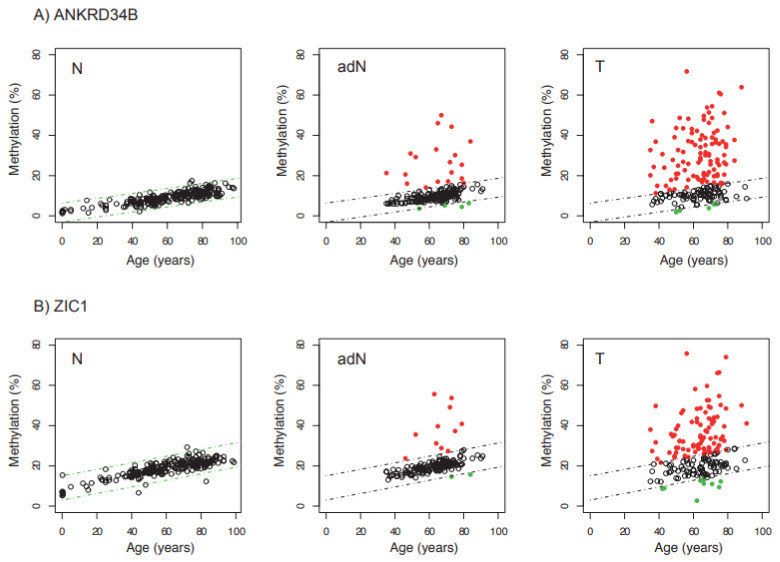
Comparison of age-dependent average methylation observed for CG1-CG7 and CG1-CG3 sites in the *ANKRD34B* (**A**) and *ZIC1* (**B**) candidate gene regions for normal (N), tumor adjacent normal (adN) and tumor (T) kidney tissue samples presenting methylation within 99% prediction channels (dashed lines) of linear regression (open black circles), above upper (solid red circles) and below lower boundaries (green circles).

DNAme is a known risk factor for tumorigenesis and measurably responds to various cancer-related lifestyle factors, such as age, inflammation, or contact with harmful substances. Therefore, we asked whether a difference in risk exposure other than age can be detected as altered mean group methylation in normal tissues with a different renal cancer risk. A comparison of 214 normal tissues with the average population risk for renal cancers and 157 (*ANKRD34B*) or 145 (*ZIC1*) adN tissue samples (i.e., cases with high cancer risk) revealed not only a clear age-dependent methylation pattern in normal and adN tissue, but also demonstrated methylation levels beyond the 99% prediction level of the linear regression, as defined by the analysis of low-risk normal tissues (Figure 3A,B: N and adN tissues). This effect was observed for the averaged methylation values of both *ANKRD34B* and *ZIC1,* and cannot be explained by age. Highly variable methylation independent of age was seen in tumor tissues (Figure 3A,B: T tissues).

**Figure 4:**
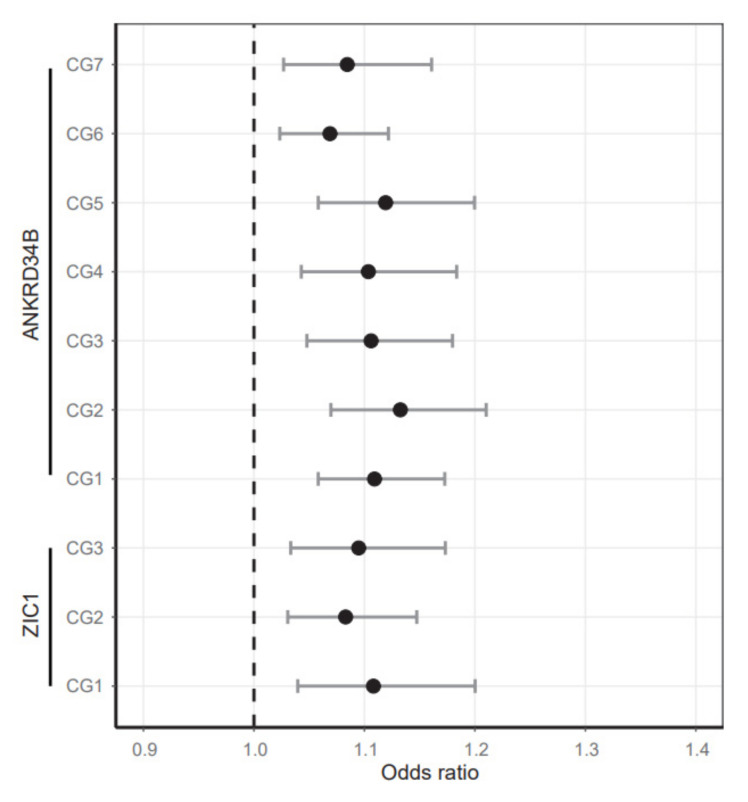
Analysis of age-independent tumor risk for CG1-CG7 and CG1-CG3 sites in the *ANKRD34B* and *ZIC1* candidate gene. OR (black filled circles) and 95% confidence intervals and the no risk OR = 1 line (dashed line) are presented. Corresponding *p*-values of logistic regression and covariate parameters of analyses are shown in Table 2.

CpG loci-specific comparisons of methylation in normal and adN tissues using logistic regression analysis revealed significant differences (Table 2, Figure 4). Odds ratios of 1.06–1.13 per 1% change in methylation were found for 9 out of 10 CpG sites located in two different gene regions (all *p* < 0.03, Bonferroni-adjusted for multiple testing). Moreover, logistic regression showed that methylation differences between tissues were not statistically explained by the age or sex of tissue donors, which turned out to be insignificant parameters in the corresponding statistical model (Table 2).

### 2.5. Analysis of CpG-Specific Methylation in Paired Tumor-Adjacent and Tumor Tissue Samples Shows Heterogeneous Alterations

The CpG site-specific comparison of paired tumor and adN tissues demonstrated tumor-specific hypermethylation for all loci (Table 3). Mean differences of 6.2–11.4% in the relative methylation, corresponding to *p*-values of 3.38 × 10^−5^–1.46 × 10^−11^, were obtained for the 10 analyzed candidate loci (Table 3).

**Figure 5:**
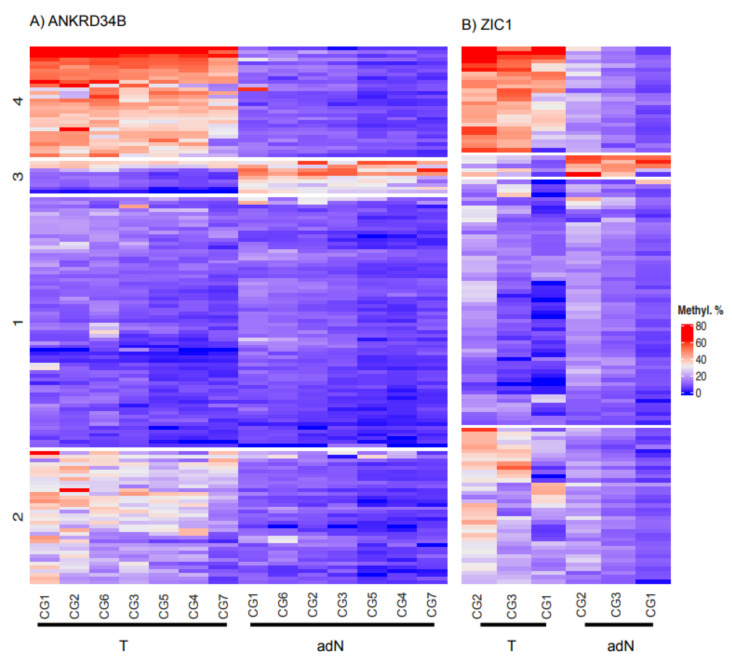
Heatmap presentation of kmeans clustering of CpG site specific methylation in paired tumor adjacent normal and tumoral tissues for independent clustering’s of *ANKRD34B* (**A**) and *ZIC1* (**B**) measured tissues. Relative methylation values were color coded as indicated (Methyl. %) and numbers refer to the designated clusters. Indicated clusters represent consensus clusters after 100 runs using bootstrapping.

A detailed comparison of CpG site-specific methylation values using a heatmap presentation of a cluster analysis demonstrated four stable patient clusters of similar size for both candidate genes (Figure 5). These included a large neutral cluster without substantial methylation changes (Cluster 1), two clusters with moderate and strong tumor-specific hypermethylation (Clusters 2 and 4), and a small cluster exhibiting hypomethylation.

### 2.6. Analysis of Human Cancer Cell Lines

**Figure 6:**
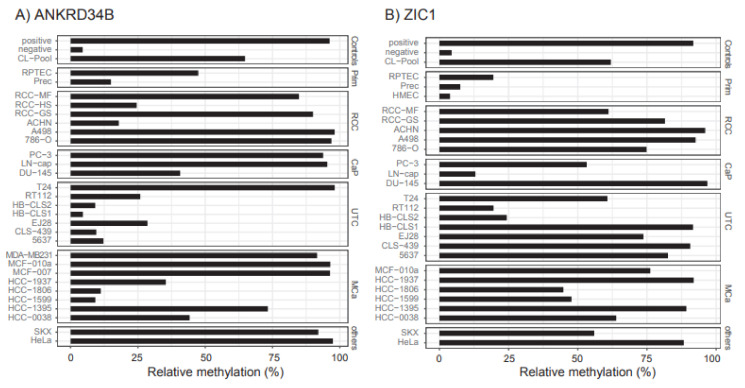
(**A**) *ANKRD34B*; (**B**) *ZIC1*. Analysis of candidate region methylation in various human tumor cell line models and controls. Designated human tumor cells were grouped according to tumor origins (RCC, renal cell cancer, CaP, prostate cancer, UTC, urothelial tumor cells, MCa, breast cancer and others).

Common cell line models of prostate, breast, urothelial, renal, and other cancers were used to estimate whether hypermethylation of *ANKRD34B* and *ZIC1* can occur with relevant frequency in human tumors. Gene-wise averaged relative methylation levels superseding the values observed for primary normal cell controls of kidney (RPTEC), prostate (Prec), and breast (HMEC) tissues were found for at least half of the kidney, prostate, urothelial, and breast cancer cell line models for both the *ANKRD34B* and *ZIC1* candidate regions (Figure 6).

## 3. Discussion

We recently described that candidate loci for age-dependent methylation can be identified by biometrical analysis of the TCGA-KIRC DNAme data using a subset of normal tumor-adjacent kidney tissues [11]. Here, we evaluated further candidate loci in *ANKRD34B* and *ZIC1* for age-related methylation and their possible additional age-independent contribution to the risk of RCC development.

Analysis of the normal autopsy tissue cohort revealed a strong association of the methylation of each of the 10 CpG sites with age. Thus, from a statistical perspective, age could explain a maximum of 77% of methylation variance (R = 0.88) in the *ZIC1*–CG2 locus. This resembles the results we previously obtained for the analysis of age-dependent TBR1 methylation in normal autopsy tissues (R = 0.85) [11]. Moreover, 9 out of 10 *ANKRD34B* sites clearly had higher coefficients of correlation compared to loci reported previously to be usable for saliva-based determination of donor age [42].

Assessment of the annual increase in age-dependent methylation showed an approximately 60% higher value for the *ZIC1*–CG2 locus (0.26% annual methylation increase) compared to the *ANKRD34B*–CG1 site (0.16% annual methylation increase). Few data have been reported on the average annual increase in normal solid tissue methylation. Previous work found an annual methylation increase of 0.06% (*SFRP1*), 0.15% (*RASSF1),* and 0.25% (*TBR1*) [10,11,43]. Overall, these results clearly identified the *ANKRD34B* and *ZIC1* regions as targets of age-related methylation in normal kidney cells, and our preliminary statistical model for predicting chronological age identified a subset of loci as possible contributors to methylation-based chronological age prediction in solid normal renal tissue (e.g., for forensic purposes). This could be of use for determining age in forensic tasks, considering the good post-mortem stability of kidney tissues for DNA-based measurements [44].

Notably, the analysis of age-dependent methylation in normal tumor-adjacent tissue samples revealed a substantial number of samples with methylation above the 99% prediction interval, as defined by the regression analysis of normal autopsy tissue samples. Levels of DNAme resembled those detected in tumor tissues and are not reached in normal control tissues even within a 100-year lifetime. Independent CpG site-specific statistical analysis using logistic regression analysis considering age and sex as covariates also demonstrated significantly increased methylation levels at 90% of the measured CpG sites in *ANKRD34B* and *ZIC1* in the high RCC-risk normal tissue group. Therefore, our results showed that additional age-independent methylation accelerators likely exist in a subset of normal tissues in the risk group. Moreover, with concurrent tumor-specific hypermethylation of loci, the corresponding CpG sites represent candidates for epigenetic renal cancer susceptibility.

Our cluster analysis of the individual DNAme of paired adN and tumor samples revealed stable groups of similar size for both candidate regions. A large sample cluster representing approximately half of the samples presented were without significant changes in DNAme, whereas two clusters were found with moderate and strong tumor-specific increases in *ANKRD34B* and *ZIC1* methylation, supporting a possible contribution of alterations in RCC development. Taking into consideration that both regions demonstrate hypermethylation in a large number of cell line models of important human cancers, these findings conceivably have a broader relevance and, in the case of *ZIC1*, this has already been confirmed in the literature [18,19,20,21,22,32,33,35,36,37,38].

On the other hand, a small cluster apparently revealed tumor-specific hypomethylation of *ANKRD34* and *ZIC1* loci starting from considerably increased methylation in adN samples, although a further increase in the tumor samples would have been expected. However, pyrosequencing only provides the detection of sample average methylation levels and, therefore, may be limited when spatial and/or cellular heterogeneity likely affects the interpretation of DNAme in individual samples. An appropriate study design and methylation detection technique are required to demonstrate a possible epigenetic lineage of tumor cells, such as that indicated by a sequence of risk factor-increased methylation of *ANKRD34B* and/or *ZIC1* in normal cells and a further increase in derived tumor cells.

Epigenetic cancer age-independent susceptibility in normal renal tissues was found previously for the *SFRP1* region [10], but could not be detected in *TBR1* loci, although both genes demonstrated clear age-dependent methylation. Thus, the newly identified *ANKRD34B* and *ZIC1* cancer loci double the number of age-independent susceptibility loci detected in normal kidney tissue, and strengthen the hypothesis that more as yet unidentified loci may exist. The epigenetic cancer risk data obtained using normal solid target tissues are thin, and most EWASs have been focused on the analysis of surrogate material, using blood samples to a large extent [3].

The present study differs from approaches making use of methylation alteration detection in blood samples for the set-up of a tissue-independent determination of epigenetic age of individuals in one essential aspect. While different variants of such epigenetic clocks have been used for statistical risk assessment of a variety of malignant and non-malignant diseases through a comparison of real and epigenetic age, the informativity for cancer risk estimation could be limited, particularly when considering the known tissue specificity of DNAme [ibid]. Thus, our approach of using normal target tissue measurement for detecting DNAme-associated cancer risk clearly circumvents the principal difficulties associated with the measurement of surrogate tissues, bearing in mind that DNAme and accumulation is assumed to be largely tissue-specific [45,46].

Notably, the ideal approach of measuring normal target tissues at risk in a prospective study design has been reported using normal cervical cells for epigenetic cancer risk prediction, likely aided by the availability of normal cells in the clinical routine [47]. However, considering that normal renal cell DNAme detection can also be carried out in principle with urine samples [48], comparable approaches for RCC prediction seem to be possible—at least theoretically.

On the one hand, the use of normal high-risk samples derived from donors with proven malignancy means that the risk-event cancer has already occurred and, theoretically, false-positive results may be obtained by tumor cell contamination of normal target tissues. On the other hand, RCC tumors normally present with a capsule that clearly separates the renal tumor mass from the normal tissues. Therefore, the gold standard surgical treatment includes laparoscopic partial resection of localized tumors, which is only possible because there are no reliable indications of the presence of extracapsular tumor cells. To the best of our knowledge, such histological evidence has not been described in the literature. Correspondingly, our histopathological evaluation of peritumoral samples, molecular analysis of CA9 tumor marker expression in adN tissues, and DNA quality of autopsy-based DNA isolation did not give any hint of either tumor cell contamination or the presence of technically associated artificial effects [10]. Moreover, the age-independent increase in normal tissue methylation was not associated with parameters of clinical aggression of the corresponding tumors, such as state of metastasis, disease progression, state of advanced disease, high-stage, or high-grade tumors (all *p* > 0.73, data not shown), which should be expected for the spread of tumor cells into surrounding normal tissue. Consequently, we consider the probability of false-positive detection of methylation in normal tumor-adjacent tissues to be extremely low.

Although the sparse functional data reported for *ANKRD34B* do not allow even hypothetical assumptions about possible causes or consequences of the DNAme of loci, a recent study collating blood and solid tissue-based EWASs aimed at investigating the effect of smoking as a lifestyle factor on DNAme identified *ZIC1* as a target in both solid human brain tissue and blood cells [39,40]. Future analyses of normal renal tissues that consider additional risk factors, such as cotinine levels in tissues and data about drug abuse, are required to systemize the search for further lifestyle factor-associated methylation that is relevant to RCC development.

## 4. Methods

### 4.1. In Silico Analysis for Candidate Identification

Candidate selection was carried out using the TCGA KIRC HM450K Illumina platform data as reported previously [11].

### 4.2. Primary Cells and Tumor Cell Lines

Analysis of primary cells and tumor cell line models was performed as described previously [11].

### 4.3. Study Design

We used a cross-sectional study design to analyze a possible relationship between DNAme of candidate loci and the age of tissue donors, each consisting of 214 normal renal autopsy and a maximum of 157 normal tumor-adjacent tissue measurements (Table 4). A case-control study comparing normal tumor-adjacent samples (cases) with normal autopsy samples (controls) was carried out to estimate the odds ratios associated with DNAme in candidate loci. Tumor-specific hypermethylation was investigated in 143 (*ANKRD34B*) and 125 (*ZIC1*) paired normal tumor-adjacent and tumor samples (Table 4).

### 4.4. Analysis of DNA Methylation

Histological and molecular evaluation of tissue samples subjected to DNA extraction, conversion of DNA, primer design, and subsequent pyrosequencing has been described previously [11,49,50]. The primer sequences used for pyrosequencing (5’- 3’) of candidate loci were: *ANKRD34B*, GAGGGATAAGGATTGGAGGAGTTAAAG (forward), Biotin-ACCCAAAAAAACCAACAACTACTAAA (reverse), TGTAGTTGTTGTTGGTTAAG (sequencing); *ZIC1*, AATAGGAGTGAGGAGAGATAGG (forward), Biotin-CCAATTTCCTTTACTTTTTCTCTCTCTTAC (reverse), AGGAGAGATAGGGTT (sequencing). The candidate loci and CpG sites measured by pyrosequencing *ANKRD34B* (CG1-CG7) and *ZIC1* (CG1-CG3) are summarized in Table 1. The genomic localization of measured and candidate CpG sites is presented in Figure 1.

### 4.5. Statistical Analysis

Age-dependent methylation of candidate loci was analyzed by CpG site-specific Pearson correlation analysis. Presentation of age-dependent methylation included the mean DNAme of the two candidate genes after linear regression and calculation of the 95% confidence interval for the regression line, as well as the 99% prediction channels. The case-control comparison of independent tissue samples was analyzed by logistic regression considering age and sex as covariates. Odds ratios and 95% confidence intervals are presented. Tumor-specific hypermethylation of paired normal and tumor tissue samples was analyzed by the two-sided paired *t*-test. The sample-specific DNAme of paired adN and tumor tissue samples was compared by unsupervised kmean clustering, presenting a consensus cluster after 100 bootstrap runs as a heatmap. Clusters exhibiting Jaccard coefficients >0.8 were considered stable clusters. All statistical calculations and presentations were done using R 3.6 [51].

## 5. Conclusions

Our study identified DNAme of *ANKRD34* and *ZIC1* as new cancer susceptibility loci for RCC development. Moreover, the loci represent targets for age-dependent methylation in solid normal kidney tissues, providing a possible contribution to chronological age determination by methylation detection in solid renal tissues.

## Figures and Tables

**Table 1:** Genomic positions of CpG sites in candidate regions and results of Pearson’s correlation analysis for association of methylation and age in normal autopsy renal tissues.

Gene.	Chrom.	Pos.	HM450K	R	R^2^	p.adj*	N	Abbr
ANKRD34B	chr5	79,866,347		0.75	0.56	0	214	CG1
	chr5	79,866,354		0.72	0.52	0	214	CG2
	chr5	79,866,359		0.74	0.55	0	214	CG3
	chr5	79,866,366		0.72	0.51	0	214	CG4
	chr5	79,866,368	cg21800232	0.70	0.49	0	214	CG5
	chr5	79,866,373		0.73	0.53	0	214	CG6
	chr5	79,866,379	cg25316339	0.61	0.37	0	214	CG7
ZIC1	chr3	147,126,196		0.70	0.49	0	214	CG1
	chr3	147,126,206	cg16181396	0.88	0.77	0	214	CG2
	chr3	147,126,218		0.74	0.55	0	214	CG3

Of note, all obtained *p*-values were below the lower calculation limit of 2 × 10^−16^. Abbreviations: Chrom., chromosome; Pos., genomic position according to hg19 genome annotation; HM450K, Illumina methylation array CpG site annotation; R, Pearson’s correlation coefficient; p.adj*, Holms adjusted *p*-values for multiple testing; N, number of measurements; Abbr, CpG site designation used in manuscript and figures.

**Table 2:** Results of CpG site specific logistic regression analysis for age and sex adjusted comparison of DNA-methylation in normal autopsy (N) and tumor adjacent normal kidney tissue samples (adN).

CpG Site	Mean N	Mean adN	Diff. Mean	OR	CI Low	CI High	*p*-Val	*p*-Val Bonf.	*p*-Val Age	*p*-Val Sex	n N	n adN
ANKRD34B												
CG1	11.4	15.0	3.6	1.11	1.06	1.17	0.000	0.001	0.425	0.549	214	157
CG2	9.9	13.1	3.2	1.13	1.07	1.21	0.000	0.001	0.492	0.576	214	157
CG3	9.2	11.9	2.6	1.11	1.05	1.18	0.001	0.006	0.776	0.712	214	157
CG4	6.8	9.1	2.3	1.10	1.04	1.18	0.002	0.016	0.932	0.690	214	157
CG5	6.7	9.6	2.9	1.12	1.06	1.20	0.000	0.003	0.914	0.675	214	157
CG6	11.6	13.8	2.2	1.07	1.02	1.12	0.004	0.029	0.814	0.693	214	157
CG7	7.3	9.2	1.9	1.08	1.03	1.16	0.010	0.071	0.446	0.815	214	157
ZIC1												
CG1	14.7	16.8	2.1	1.11	1.04	1.20	0.006	0.017	0.981	0.822	214	145
CG2	22.8	25.5	2.7	1.08	1.03	1.15	0.004	0.011	0.257	0.801	214	145
CG3	18.8	20.7	2.0	1.09	1.03	1.17	0.005	0.015	0.871	0.968	214	145

CG annotation refers to genomic positions shown in Table 1. Abbreviations: Diff., difference; OR, odds ratio; CI low and high, lower and higher boundaries of 95% confidence interval; Bonf., Bonferroni adjustment for multiple testing.; n N, number of normal autopsy kidney tissues; n adN, number of tumor adjacent normal tissue samples.

**Table 3:** CpG site-specific statistical comparison of paired tumor adjacent normal and tumor tissue samples using the two-sided *t*-test.

Region.	CpG	Diff. Mean	CI Low	CI High	*p*-Value	*p*-Value (Adj.)
ANKRD34B	CG1	11.4	8.3	14.6	4.80 × 10^−11^	1.44 × 10^−10^
	CG2	11.9	8.8	15.0	2.90 × 10^−12^	1.74 × 10^−11^
	CG3	10.4	7.5	13.3	5.41 × 10^−11^	1.44 × 10^−10^
	CG4	11.1	8.3	13.9	2.09 × 10^−12^	1.46 × 10^−11^
	CG5	11.1	8.1	14.0	7.10 × 10^−12^	3.55 × 10^−11^
	CG6	10.5	7.7	13.4	1.89 × 10^−11^	7.58 × 10^−11^
	CG7	7.7	5.1	10.3	4.41 × 10^−08^	4.41 × 10^−08^
ZIC1	CG1	6.2	3.3	9.0	3.38 × 10^−05^	3.38 × 10^−05^
	CG2	8.4	5.8	11.1	3.93 × 10^−09^	1.18 × 10^−08^
	CG3	7.6	4.9	10.3	1.17 × 10^−07^	2.35 × 10^−07^

Abbreviations: Diff., difference; CI, 95% confidence interval; adj., holm adjustment for multiple testing.

**Table 4:** Tissue characteristics of normal autopsy (N), tumor adjacent normal (adN) and tumor tissues (T).

Tissue		N		adN		adN*/T	
Gene		ANKRD34B	ZIC1	ANKRD34B	ZIC1	ANKRD34B	ZIC1
Total (n)		214	214	157	145	143	125
Age (y)	Median (min–max)	61 (0–98)	60 (0–99)	66 (35–91)	65 (35–91)	66 (35–91)	66 (35–91)
Sex (n)	female (%)	77 (36.0)	72 (33.6)	58 (36.9)	53 (36.6)	53 (37.1)	46 (36.8)
	male (%)	137 (64.0)	142 (66.4)	99 (63.1)	92 (63.4)	90 (62.9)	79 (63.2)

Note that Total (n) of the cohort refers to the measurement of largely, but not perfectly, equal subsets of a larger cohort of 239 N samples. adN* samples represent a subset of adN samples with available paired tumor tissue measurements.

## Data Availability

The anonymized datasets used and/or analyzed during the current study are available as supplementary materials.

## Supplementary Materials

The following supporting information can be downloaded at: https://www.mdpi.com/article/10.3390/ijms23105327/s1.
