# Supplementary material for: Age-Related DNA Methylation in Normal Kidney Tissue Identifies Epigenetic Cancer Risk Susceptibility Loci in the ANKRD34B and ZIC1 Genes
Source: Int J Mol Sci. 2022 May 10;23(10):5327. doi: 10.3390/ijms23105327 (PMC9141100; doi:10.3390/ijms23105327)
Supplement: Supplementary file 1 [file ijms-23-05327-s001.zip › Suppl_Fig_Captions.pdf]

Suppl. Figure S1 Exemplary presentation of unprocessed pyrosequencing data for *ANKRD34B* (A) and *ZIC1* (B) candidate loci showing unmethylated DNA (negative control, nc) and fully methylated DNA (positive control, pc). Shaded areas mark variable positions used by the Pyromark software for calculation of relative methylation of the corresponding CpG sites.

Suppl. Figure S2 Residuals plot of the chronological age prediction model using *ANKRD34B* and *ZIC1* loci. Predicted chronological age (open circles) is plotted against real age (solid black circles).
