# Supplementary figures and images for: Age-Related DNA Methylation in Normal Kidney Tissue Identifies Epigenetic Cancer Risk Susceptibility Loci in the ANKRD34B and ZIC1 Genes
Source: Int J Mol Sci. 2022 May 10;23(10):5327. doi: 10.3390/ijms23105327 (PMC9141100; doi:10.3390/ijms23105327)

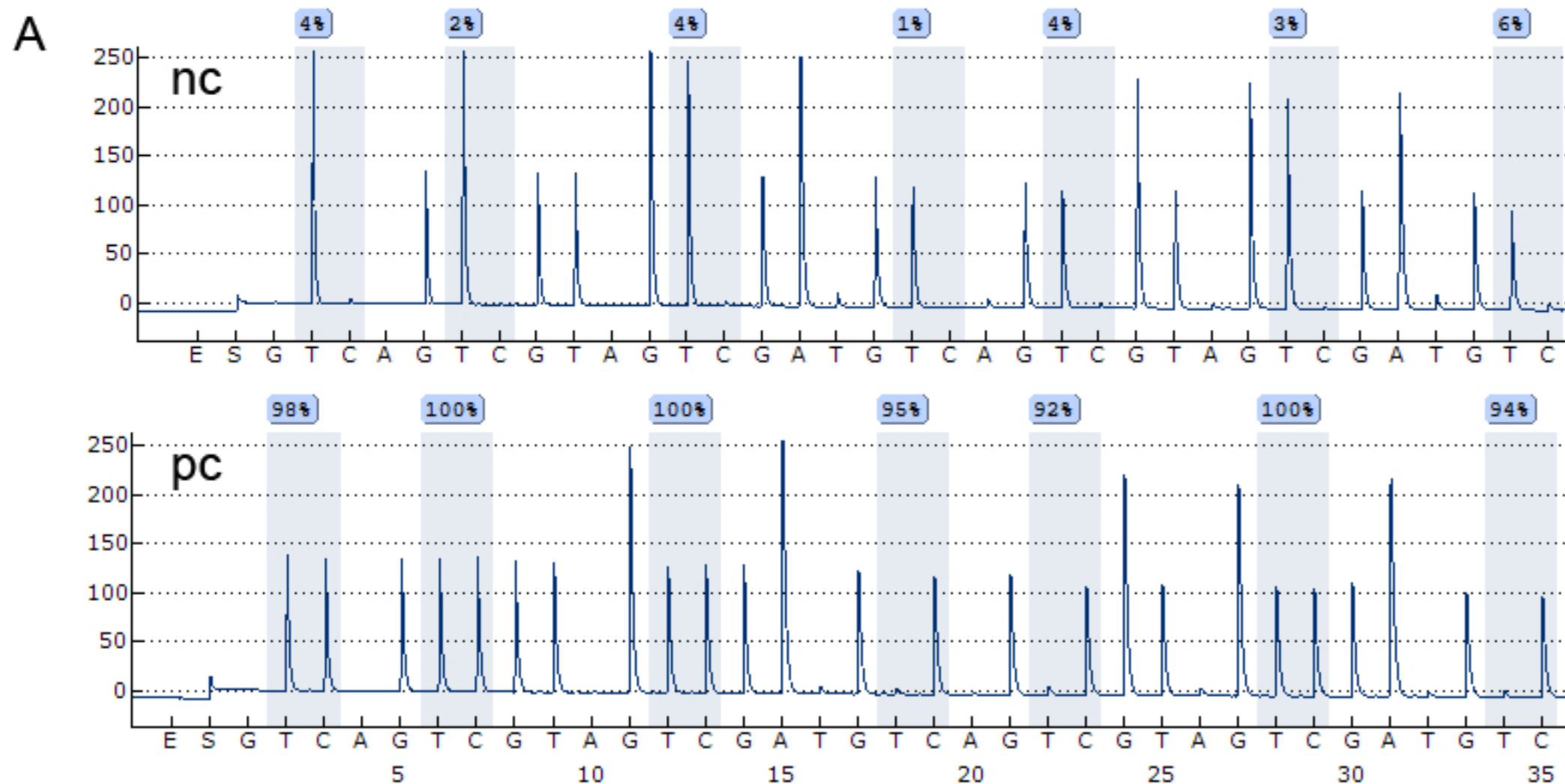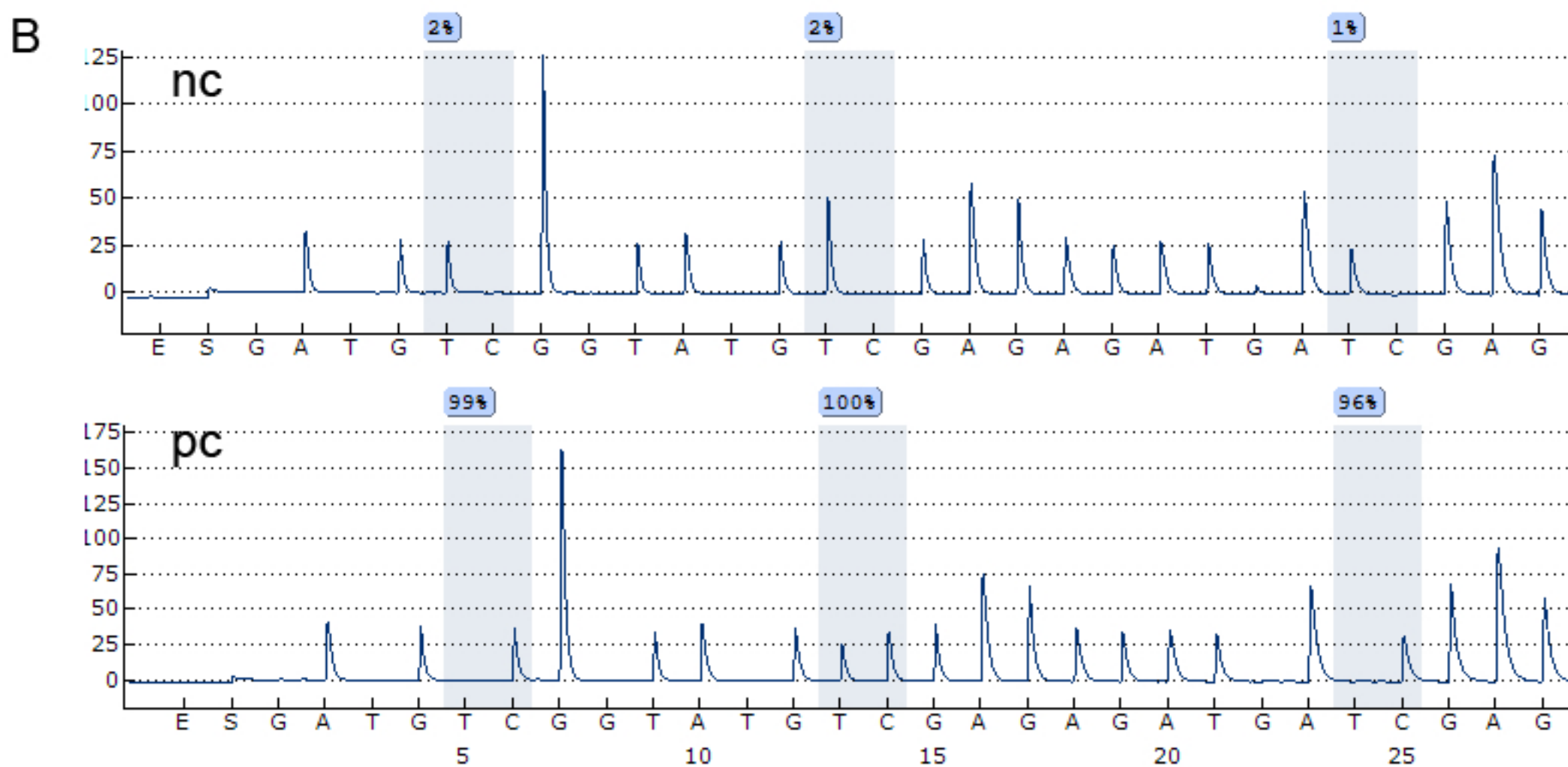

Supplement: Supplementary file 1 [file ijms-23-05327-s001.zip › Suppl Figure S1.pdf]

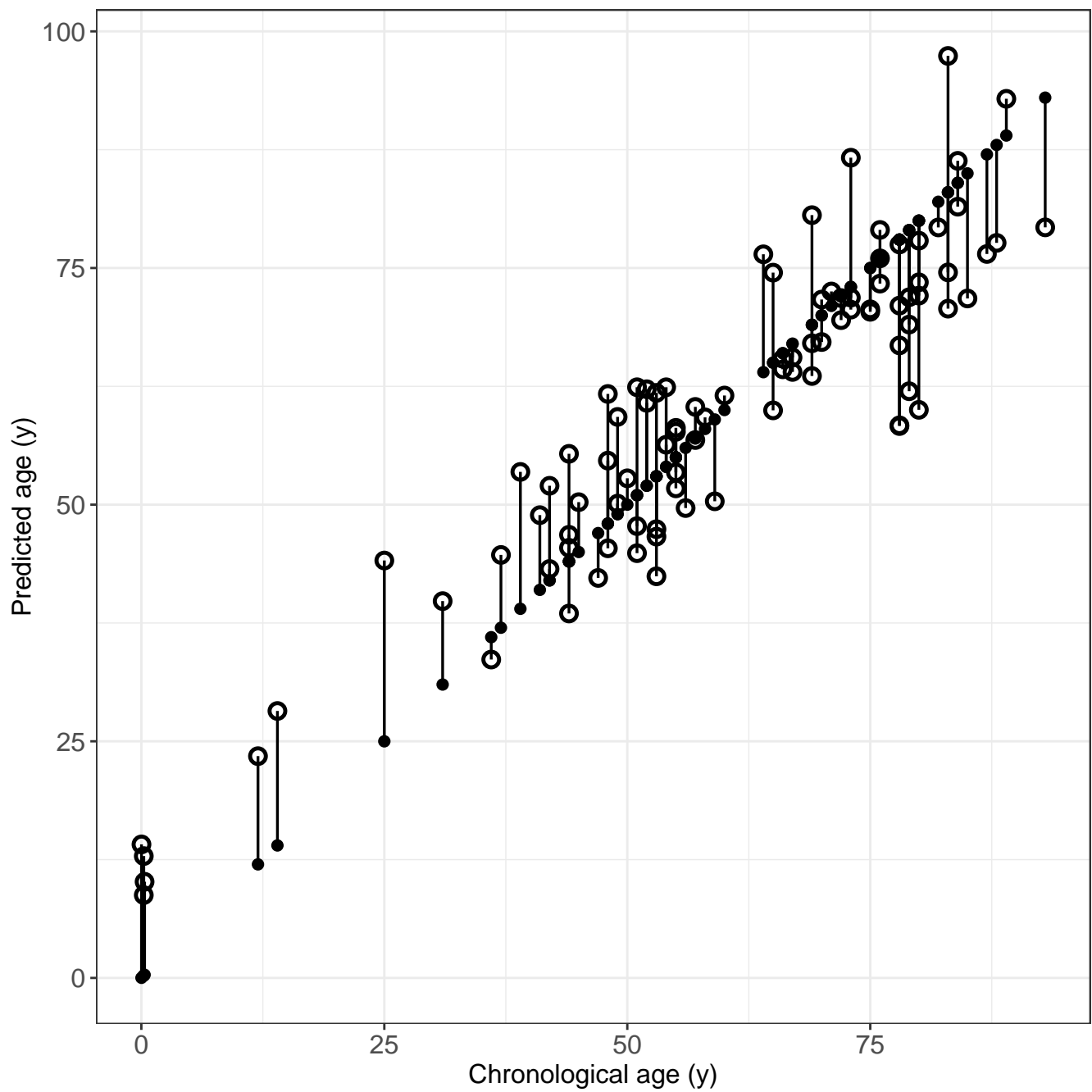

Supplement: Supplementary file 1 [file ijms-23-05327-s001.zip › Suppl Figure S2.pdf]
